# Radiofrequency Electromagnetic and Pulsed Magnetic Fields Protected the Kidney Against Lipopolysaccharide-Induced Acute Systemic Inflammation, Oxidative Stress, and Apoptosis by Regulating the IL-6/HIF1α/eNOS and Bcl2/Bax/Cas-9 Pathways

**DOI:** 10.3390/medicina61020238

**Published:** 2025-01-29

**Authors:** Çağrı Balci, Mustafa S. Özcan, Halil Aşci, Pınar Karabacak, Oya Kuruşçu, Rümeysa Taner, Özlem Özmen, Muhammet Y. Tepebaşi, İlter İlhan, Selçuk Çömlekçi

**Affiliations:** 1 Department of Anesthesiology and Reanimation, Faculty of Medicine, Suleyman Demirel University, 32260 Isparta, Turkey; cagribalci@sdu.edu.tr (Ç.B.); pinarkarabacak@sdu.edu.tr (P.K.); oyakuruscu@sdu.edu.tr (O.K.); 2 Department of Pharmacology, Faculty of Medicine, Suleyman Demirel University, 32260 Isparta, Turkey; halilasci@sdu.edu.tr; 3 Department of Bioengineering, Institute of Science, Suleyman Demirel University, 32260 Isparta, Turkey; rumeysataner21@gmail.com (R.T.); scom56@gmail.com (S.Ç.); 4 Department of Pathology, Faculty of Veterinary Medicine, Burdur Mehmet Akif Ersoy University, 15200 Burdur, Turkey; ozlemoz@mehmetakif.edu.tr; 5 Department of Medical Genetics, Faculty of Medicine, Suleyman Demirel University, 32260 Isparta, Turkey; muhammettepebasi@sdu.edu.tr; 6 Department of Biochemistry, Faculty of Medicine, Suleyman Demirel University, 32260 Isparta, Turkey; ilterilhan@sdu.edu.tr; 7 Department of Electronics and Communication Engineering, Faculty of Engineering, Suleyman Demirel University, 32260 Isparta, Turkey

**Keywords:** acute kidney injury, inflammation, pulsed magnetic field (PMF), radiofrequency electromagnetic field (RF-EMF), sepsis

## Abstract

*Background/Objectives*: Sepsis-associated acute kidney injury caused by lipopolysaccharide (LPS) is related to hypoxia, amplification of the inflammatory response, oxidative stress, mitochondrial dysfunction, and apoptosis. This study aims to explore the protective effects of a radiofrequency electromagnetic field (RF-EMF) and a pulsed magnetic field (PMF) on acute kidney injury in rats. *Materials and methods*: Forty female Wistar albino rats were randomly divided into five groups (each containing eight rats): control, LPS, RF-EMF, PMF, and RF-EMF + PMF groups. Six hours after LPS application, blood and tissues were removed for histopathological, immunohistochemical, biochemical, and genetic analysis. *Results*: Histopathological findings, caspase-3, inducible nitric oxide synthase and tumor necrosis factor-alpha immunoexpressions, total oxidant status and oxidative stress index levels, and interleukin-6, hypoxia-inducible factor alpha, Bcl-2-associated X protein, and caspase 9 gene expression in kidney tissue and blood urine nitrogen and creatinine levels in blood were increased, whereas endothelial nitric oxide synthase and B-cell lymphoma 2 gene expression were decreased in the LPS groups. Both RF-EMF and PMF reversed all these findings and recovered renal tissues. *Conclusions*: Noninvasive, nontoxic, low-cost PMF and RF-EMF, both single and combined, have been demonstrated to have renoprotective anti-inflammatory, antioxidant, and antiapoptotic effects.

## 1. Introduction

Sepsis is characterized by a dysregulated host response leading to life-threatening organ dysfunctions, including acute kidney injury (AKI) [1]. Kidneys are very susceptible to damage during sepsis because of their high perfusion, and are among the earliest affected organs [2]. Sepsis-associated acute kidney injury (SA-AKI) has a poor prognosis and is related to increased mortality and length of hospitalization [3]. Despite advances in our current understanding of the pathophysiology of sepsis, millions of people still die each year. Therefore, current treatment practices appear to be inadequate and limited, so it is crucial to investigate new treatment modalities [4,5].

Mechanisms such as microcirculatory disruption, hypoxia, amplification of the inflammatory response, oxidative stress, mitochondrial dysfunction, and apoptosis play important roles in the pathogenesis of SA-AKI [3,4]. It is crucial to clarify these mechanisms for the treatment of sepsis. Inflammatory cytokines such as interleukin-6 (IL-6) affect the expression and activity of hypoxia-induced factor alpha (HIF-1α), endothelial nitric oxide synthase (eNOS), and nicotinamide adenine dinucleotide phosphate (NADPH) oxidase, thereby affecting nitric oxide (NO) and superoxide levels and contributing to oxidative stress [6,7,8]. Apoptosis is mediated by the activation of enzymes called caspases (Cas). Caspase activation depends on a delicate balance between the production and degradation of proapoptotic and antiapoptotic proteins. Among these proteins, B-cell lymphoma 2 (Bcl-2), Bcl-2-associated X protein (Bax), and Cas-9 are essential for indicating mitochondrial apoptosis [5,9].

Many studies on various experimental models have demonstrated the beneficial effects of electromagnetic applications on multiple tissues [10,11,12,13,14,15]. Electric fields (EFs) and radiofrequency electromagnetic fields (RF-EMFs) influence biological systems through various mechanisms that are highly sensitive to exposure parameters. Small variations in frequency, intensity, or duration can produce drastically different biological outcomes [16]. Pulsed magnetic field (PMF) and RF-EMF applications can cause low-level and harmless biochemical changes in cells when used at appropriate power and frequency [10,17,18,19]. PMF and RF-EMF treatment independently appear to have significant potential to reduce inflammation in cells through multiple mechanisms, including cytokine modulation, inhibition of key inflammatory pathways, reduction in oxidative stress, and modulation of immune cell activity [18,19,20,21,22]. They may show additive or synergistic effects when used in combination. There are almost no studies on the effects of PMF and RF-EMF in sepsis models, either alone or in combination. Based on the results of previous studies in different models, we hypothesized that these applications could be used in individuals with sepsis [10,23].

This study aims to investigate the effects of RF-EMF and PMF therapies on kidney injury in a lipopolysaccharide-induced acute sepsis model in rats and to elucidate the mechanism of action through the IL-6/HIF-1α/eNOS and Bcl2/Bax/Cas-9 pathways.

## 2. Materials and Methods

### 2.1. Magnetic Field Applications

**Figure 1:**
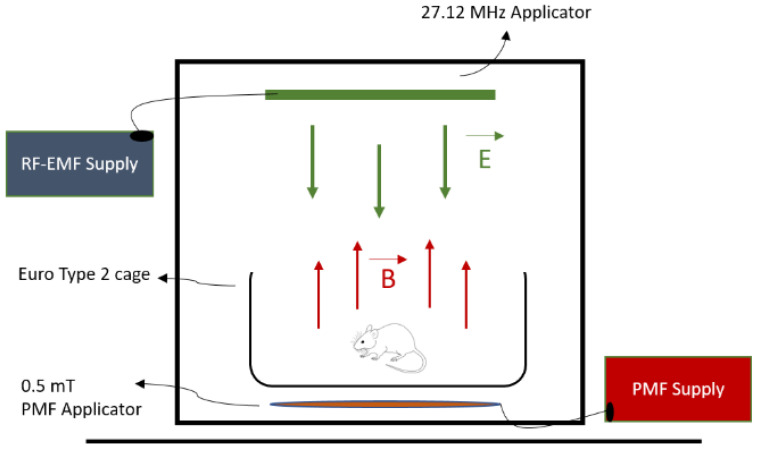
RF-EMF and PMF unit diagrams.

A representative technical drawing of the experimental setup used in the study is given in Figure 1. A digital Gauss meter was used to measure the magnetic field strength (Unilab Digital Gauss/Tesla meter, Blackburn, UK). The RF-EMF energy source in the setup was subjected to a preliminary test. In accordance with the project objectives, energy with a value of approximately 1 W antenna output was positioned so that the directly parallel connected printed circuit board (PCB) antenna would face the rat’s surface.

In such PCB antenna setups used in other studies by the team, the PCB antenna + oscillator at a frequency of 27.12 MHz creates an electric field of 10 V/m at a distance of 15 cm with a 0.8 Watt RF-EMF output power under a 12 Volt DC supply. The effect of this frequency RF-EMF energy, which has passed the FDA’s approval, starts at this threshold value. Therefore, an electric field value of 10 V/m was selected. In the same experimental setup, for a PMF that is as homogeneous as possible, a working value below the 1 mT limit value recommended by the World Health Organization for the United States was used [24]. For the 0.5 mT, 1 Hertz monophasic square-wave PMF used in our team’s previous studies, the magnetic field intensity value of 0.5 mT from a distance of 15 cm was selected as the target; in other words, while the current value at which the PMF source drives the inductive applicator was calculated, the current value that creates a homogeneous 0.5 mT magnetic field value in the desired region under realistic conditions of the experiment was measured. Intermittent 12 V voltages were obtained from a laboratory-type power supply supplied by a 220 V main voltage. However, the power supply was not directly connected to the 220 V mains; first, a custom-made 1 s flasher circuit was connected to the mains. The flasher circuit output feeds the power supply. Thus, the power supply fed at 1 s intervals produces a 1 s 12 V voltage.

The power supply feeds the coils for 1 s and does not feed them for 1 s. Thus, the coils produce 1 s magnetic field pulses. There are two PCB antennas with a value of 27.12 MHz for the RF-EMF system. These two PCB antennas are placed at the top of the unit and at the bottom of the unit where two coil cages are prepared using an enameled winding wire for PMF are placed. The system was prepared by connecting the RF-EMF and PMF applicators to the power source. A radio frequency EMF strength meter (Extech 480836, Nashua, NH, USA) spectrum analyzer was used to measure RF-EMF exposure in the environment during the experiment. ELF meter PF-4 (Extech 480836, Lancashire, UK) was used for PMF exposure (Figure 1).

### 2.2. Ethical Approval and Animals

The processes and procedures intended to be performed on rats were reviewed and approved by the Local Ethics Committee of Süleyman Demirel University Animal Experiments (Ethics No: 01.11.2023/10-222). All experiments were performed per the Animal Research: Reporting in Vivo Experiments (ARRIVE) guidelines, version 2.0 protocol. In addition, this study was supported by the Scientific Research Projects Coordination Unit of Suleyman Demirel University with the project code TTU-2024-9251 and the Scientific and Technological Research Council of Turkey (TUBİTAK) (project code: 223S402).

The sample size was determined using power analysis, with a type 1 error (α) of 0.05, a type 2 error (β) of 0.20 (power = 0.80), effect size of 0.6, and a minimum of 8 animals per group. A total of 48 adult female Wistar albino rats (8 for the preliminary study, 40 for the main study) weighing 250–300 g and aged of 12–14 weeks were obtained from the Suleyman Demirel University Animal Experiments Laboratory. Rats in the same estrous cycle were included in this study using vaginal smear to ensure standardization of the study. Each group of rats was housed in separate standard Euro-type 4 cages. The study environment was carefully regulated to maintain a temperature range of 21–22 °C and a humidity level of 55–65%. In addition, all of the rats were kept under a consistent 12 h light/12 h dark cycle as part of the experimental conditions.

### 2.3. Experimental Procedures

A preliminary study was conducted by creating a lipopolysaccharide (LPS)-induced acute kidney injury model to determine the most appropriate duration for both magnetic field applications used in the experiment.

Eight female Wistar albino rats were randomly divided into 8 groups as controls: the LPS (5 mg/kg), PMF (0.5 h/3 h/6 h), and RF-EMF (0.5 h/3 h/6 h) groups (each containing one rat). For the induction of sepsis, a single dose of 5 mg/kg ip LPS was used. Six hours after LPS administration, the rats in all the groups were sacrificed under ketamine (80–100 mg/kg) and xylazine (8–10 mg/kg) anesthesia and euthanized via the surgical exsanguination method. The left renal artery, vein, and kidney tissues of each animal were placed in 10% formaldehyde for histopathological analysis.

According to the histopathological findings of the preliminary study, the kidneys of the control rats were normal. Hyperemia and microhemorrhages were observed in the kidneys of LPS-applicated rats. In the histopathologic examination of the kidney tissues of the rats treated with the PMF, hyperemia and microhemorrhages persisted in the 0.5 h application. Pathologic findings were observed to improve in the 3 h application. Six-hour application did not cause a significant increase in the improvement of pathological findings and caused mild hyperemia. In the histopathological examination of the kidney tissues of rats treated with the RF-EMF, it was noted that 0.5 h application significantly improved the pathological findings caused by LPS. However, severe hyperemia was observed in the kidneys after 3 and 6 h of treatment.

On the basis of these findings, the optimal application time was found to be a 3 h duration for PMF and a 0.5 h duration for RF, resulting in a marked improvement. Therefore, it was decided that the main study groups would be conducted using these optimal durations for both applications.

**Figure 2:**
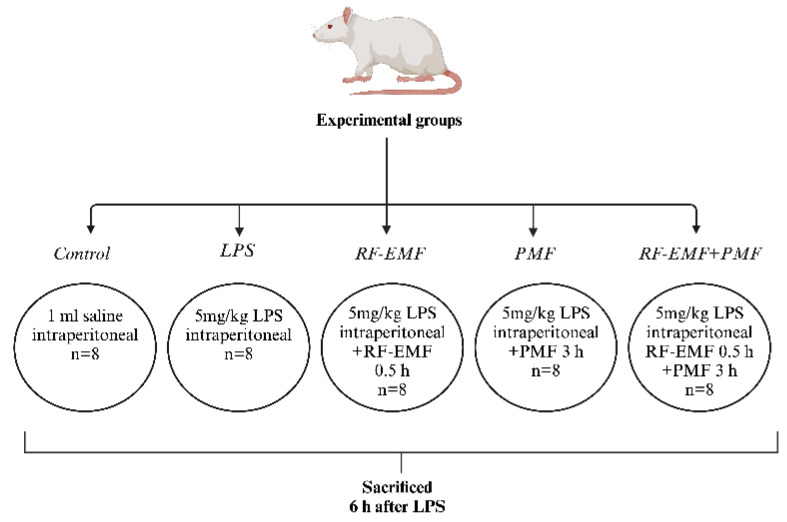
Flow diagram for experimental grouping.

In the main study, 40 female Wistar albino rats were randomly divided into 5 groups (each containing 8 rats) (Figure 2).

1. Control group: Rats that received 1 mL of i.p. saline injection to create a similar stress level were left in an inactivated unit for 6 h.
2. LPS group: Rats that received a single dose of 5 mg/kg i.p. LPS to establish an acute sepsis model were left in an inactivated unit for 6 h.
3. RF-EMF group: Rats that received a single dose of 5 mg/kg i.p. LPS were maintained in an activated RF-EMF unit for 0.5 h. Then, they were left in an inactivated unit until the 6th hour.
4. PMF: Rats that received a single dose of 5 mg/kg i.p. LPS were maintained in an activated PMF unit for 3 h. Then, they were left in an inactivated unit until the 6th hour.
5. RF-EMF + PMF: To create an acute sepsis model, rats that received a single dose of 5 mg/kg i.p. LPS were subjected to a combination of simultaneously activated RF-EMF (for 0.5 h) and PMF (for 3 h) applications. The rats were left in the inactivated unit until the 6th hour.

Six hours after LPS application, the rats in all the groups were sacrificed under ketamine (80–100 mg/kg) and xylazine (8–10 mg/kg) anesthesia. Following abdominal incision, euthanasia was performed using surgical exsanguination with blood taken from the vena cava inferior. After the blood samples were centrifuged at 3000 rpm for 10 min (min), they were transferred to the biochemistry laboratory for analysis of blood urine nitrogen (BUN) and creatinine levels via a spectrophotometric method.

The left renal artery and kidney tissues of each animal were preserved in 10% formaldehyde for histopathological hematoxylin and eosin staining and immunohistochemical analyses of the inflammation indicators such as tumor necrosis factor-alpha (TNF-α), Cas-3, and inducible nitric oxide synthesis (iNOS). Half of the right kidney tissues were stored in −20 °C refrigerators to examine the total oxidant status (TOS) and total antioxidant status (TAS) oxidative stress parameters, and the remaining tissues were stored in −80 °C refrigerators to perform genetic analyses of IL-6, HIF-1α, eNOS, Bcl-2, Bax, and Cas-9 expression.

### 2.4. Histopathological Examination

At the end of the study, all of the rats from each group were euthanized, and kidney tissue samples were collected during necropsy. For histopathological and immunohistochemical examination, the tissue samples were placed in a 10% buffered formaldehyde solution. After being fixed in formaldehyde for two days, the cassettes were processed via a fully automated tissue processor (Leica ASP300S; Leica Microsystem, Nussloch, Germany) for routine histopathological tissue processing. The next morning, paraffin blocks of the kidney tissue samples were prepared. After the blocking process, serial 5 µm thick sections were obtained from the blocks using a fully automated Leica 2155 microtome (Leica Microsystem, Nussloch, Germany). All of the sections were then stained with hematoxylin-eosin (HE). Finally, the sections were cleared in xylene, mounted with coverslips using Entellan, and examined under a light microscope. Histopathological evaluations were performed under a 20× objective lens, with scoring based on hyperemia, hemorrhage, and inflammatory cell infiltration using a scale from 0 to 3 (Table 1). Morphometric analyses and microphotography were carried out via the Database Manual Cell Sens Life Science Imaging Software System [ver.4.1] (Olympus Co., Tokyo, Japan). The obtained results were evaluated using statistical analyses.

### 2.5. Immunohistochemical Examination

For immunohistochemical analysis, three serial sections were taken from the paraffin blocks along with the sections for histopathological examination, and these sections were mounted on poly-L-lysine-coated slides. The streptavidin-biotin complex peroxidase method was used to evaluate the expression of TNF-α (anti-TNF alpha antibody [12] (ab205587)), iNOS (recombinant anti-iNOS antibody (ab283655)), and caspase-3 (recombinant anti-caspase-3 antibody [EPR18297] (ab184787)). The sections were subjected to immunohistochemical staining in a 1/100 dilution. Primary and secondary antibodies from Abcam (Cambridge, UK) were used for this analysis.

The secondary antibody used was the EXPOSE Mouse and Rabbit Specific HRP/DAB Detection IHC Kit (ab80436), with diaminobenzidine (DAB) serving as the chromogen (Abcam, Cambridge, UK). Positive controls were included for each marker, whereas for negative controls, the primary antibody stage was omitted. Each evaluation was performed in a blinded manner. The reaction was visualized by applying 3,3′-diaminobenzidine (DAB) chromogen to the sections. Subsequently, Harris hematoxylin was used for counterstaining, completing the immunohistochemical method.

The intensity of immunohistochemical staining was semi-quantitatively scored on a scale from 0 to 3, where 0 = negative, 1 = mild expression, 2 = moderate expression, and 3 = strong expression (Table 1). Statistical analysis was conducted on the scores to assess differences between groups. ImageJ 1.46r software (National Institutes of Health, Bethesda, MD, USA) was used for immunohistochemical scoring.

### 2.6. Biochemical Examination

Kidney tissues obtained for oxidative stress parameters were portioned, placed in Eppendorf tubes, and stored at −20 °C until the day of analysis. The tissues were subsequently diluted fivefold (*w*/*v*) with phosphate-buffered saline (10 mM sodium phosphate) at pH 7.4 and homogenized via a tissue homogenizer (IKA Ultra Turrax T25, Janke & Kunkel, Staufen, Germany). After homogenization, the samples were centrifuged at 2000 rpm for 20 min at/+4 °C (Nuve NF 1200R, Ankara, Turkey). The samples were subsequently transferred to the SDU Faculty of Medicine Biochemistry Laboratory for triplicate measurements. To assess oxidative stress, the levels of TAS and TOS were analyzed via the spectrophotometric method based on Erel’s protocol. The TOS results are expressed as µmol H_2_O_2_/g protein, and the TAS results are expressed as mmol Trolox Eq/g protein. The OSI was calculated by dividing the TOS levels by the TAS levels, that is, TOS/TAS/10.

The collected rat blood was centrifuged at 3000 rpm for 15 min, and the serum urea and creatine levels were measured spectrophotometrically with a Beckman Coulter AU5800 autoanalyzer (Beckman Coulter, Brea, CA, USA).

### 2.7. Reverse Transcription-Polymerase Chain Reaction (RT-qPCR)

PCR/qPCR and clustered regularly interspaced short palindromic repeats (CRISPR)-associated systems are used to detect nucleic acid signatures of pathogenic microorganisms with varying characteristics such as speed, sensitivity, and specificity [25]. A real-time qPCR device was used in the context of the facilities of the study laboratory. Using the manufacturer’s protocol, RNA was isolated from homogenized tissues with the GeneAll RiboEx (TM) RNA Isolation Kit (GeneAll Biotechnology, Seoul, Republic of Korea). The amount and purity of the RNAs obtained were measured with a BioSpec-nano nanodrop (Shimadzu Ltd., Kyoto, Japan) device. One microgram of RNA was used for cDNA synthesis. cDNA synthesis was performed with an A.B.T.™ cDNA Synthesis Kit (Atlas Biotechnology, Ankara, Turkey) in a thermal cycler according to the manufacturer’s protocol. The primers were designed by detecting specific mRNA sequences and testing possible primer sequences via the NCBI website (Table 2). Gene expression levels were measured using a Bio-Rad CFX96 (Bio-Rad Laboratories, Inc., Hercules, CA, USA) real-time PCR instrument with 2X SYBR green master mix (Selleck Bioreagents, Kocaeli, Turkey). In this study, the GAPDH gene was used as a housekeeping gene. The reaction mixture was prepared according to the manufacturer’s protocol to a final volume of 20 µL. The resulting reaction mixture was placed in a real-time qPCR device according to the kit manufacturer’s protocol, and each sample was studied in triplicate. The following 40 cycles of RT-qPCR were used: initial denaturation at 94 °C for 10 min, denaturation at 95 °C for 15 s, and annealing/extension at 55 °C for 30 s. Relative mRNA levels were calculated by applying the 2^−ΔΔCt^ formula to the normalized results.

### 2.8. Statistical Analysis

The histopathological, biochemical, and genetic results were analyzed for normality distribution, and, since all the data were normally distributed, one-way ANOVA was used, followed by Tukey’s multiple comparison test. Statistical analysis was performed using GraphPad Prism version 8.0 software (San Diego, CA, USA). Differences were considered significant at *p*  < 0.05. All of the results are expressed as the means ± SD.

## 3. Results

### 3.1. Histopathological and Immunohistochemical Findings

**Figure 3:**
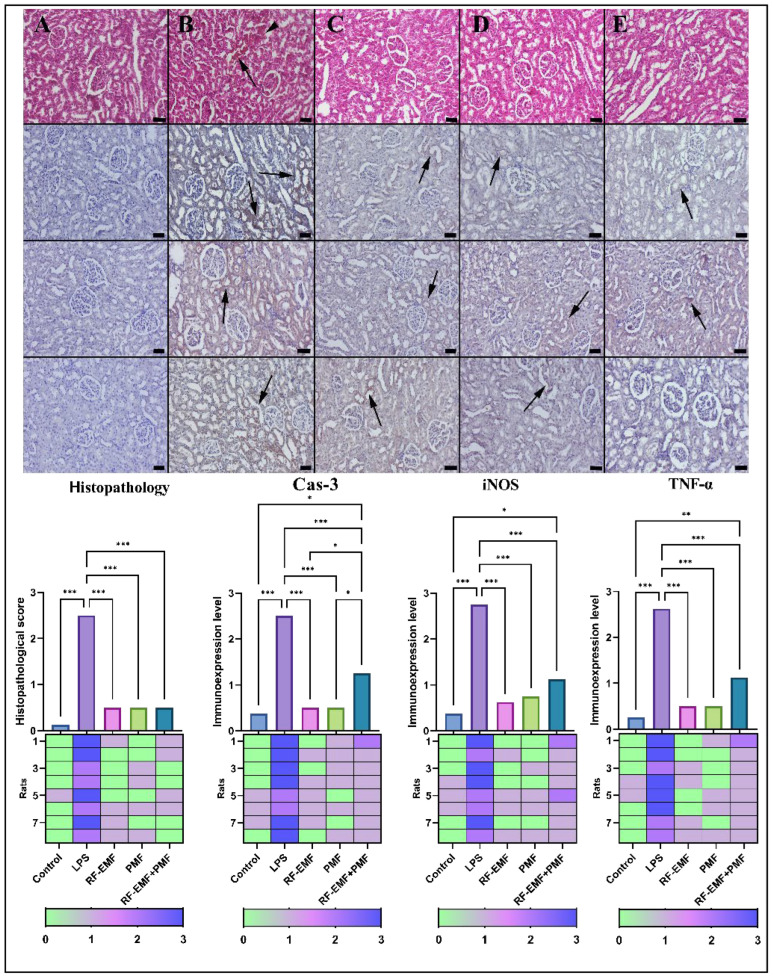
Histopathological (**upper** row) and immunohistochemical analysis of caspase (**second** row), iNOS (**third** row), and TNF-α (**fourth** row) expression in kidney tissues. (**A**) Control group: Normal kidney histology with minimum caspase-3, iNOS, and TNF-α expression. (**B**) LPS group: Severe hyperemia (arrow), microhemorrhages (arrowhead), and significantly increased caspase-3, iNOS, and TNF expression (arrows). (**C**) PMF group: Restored kidney histology with substantially reduced caspase-3, iNOS, and TNF-α expression (arrows). (**D**) RF group: Normal kidney morphology with marked decrease caspase-3, iNOS, and TNF-α expression (arrows). (**E**) Combined PMF + RF group: Normal kidney appearance and significantly lower caspase-3, iNOS, and TNF-α expression (arrows) comparable to control levels. The upper row displays HE staining, while the remaining rows demonstrate results of the streptavidin-biotin peroxidase method. Scale bars = 50 μm. Data are shown as the means ± standard deviations. Statistical significance was determined using one-way ANOVA: * *p* < 0.05, ** *p* ≤ 0.01 *** *p* ≤ 0.001.

Normal tissue histology was detected in the kidneys of the rats in the control group. Severe hyperemia and microhemorrhages were observed in the kidneys of the rats in the LPS group compared the control group (*p* < 0.001). Notably, PMF and RF-EMF applications significantly improved the pathological findings caused by LPS (*p* < 0.001). The pathological findings were similar to those of the single applications in the group in which PMF and RF-EMF were administered together (*p* < 0.001 for all) (Figure 3).

When Cas-3 expression was examined according to the groups, the negative or very mild expression in the control group increased with LPS application (*p* < 0.001). Notably, PMF and RF-EMF significantly decreased Cas-3 expression (from 2.75 ± 0.46 to 0.75 ± 0.46 and 0.62 ± 0.51, respectively) (*p* < 0.001). In addition, a decrease in expression was observed in the group in which both were administered together (*p* < 0.001 for all) (Figure 3).

When the iNOS expression in the kidneys of the groups was evaluated, negative or very mild expression was observed in the control group. In the LPS group, the expression of these genes increased significantly, especially in the tubule epithelium. Notably, the PMF and RF-EMF applications significantly reduced the iNOS expressions (from 2.5 ± 0.53 to 0.5 ± 0.53, for both). In addition, the expression of these genes was reduced in the group in which both were applied together (*p* < 0.001 for all) (Figure 3).

When the TNF-α expression in the kidneys of the different groups was examined, the expression that was negative or very light in the control group increased significantly with increasing LPS concentration (*p* < 0.001). Notably, PMF and RF-EMF significantly decreased TNF-α expression (from 2.62 ± 0.51 to 0.5 ± 0.53 for both) (*p* < 0.001). The expression was reduced in the group in which both were applied together (*p* < 0.001 for all) (Figure 3).

**Figure 4:**
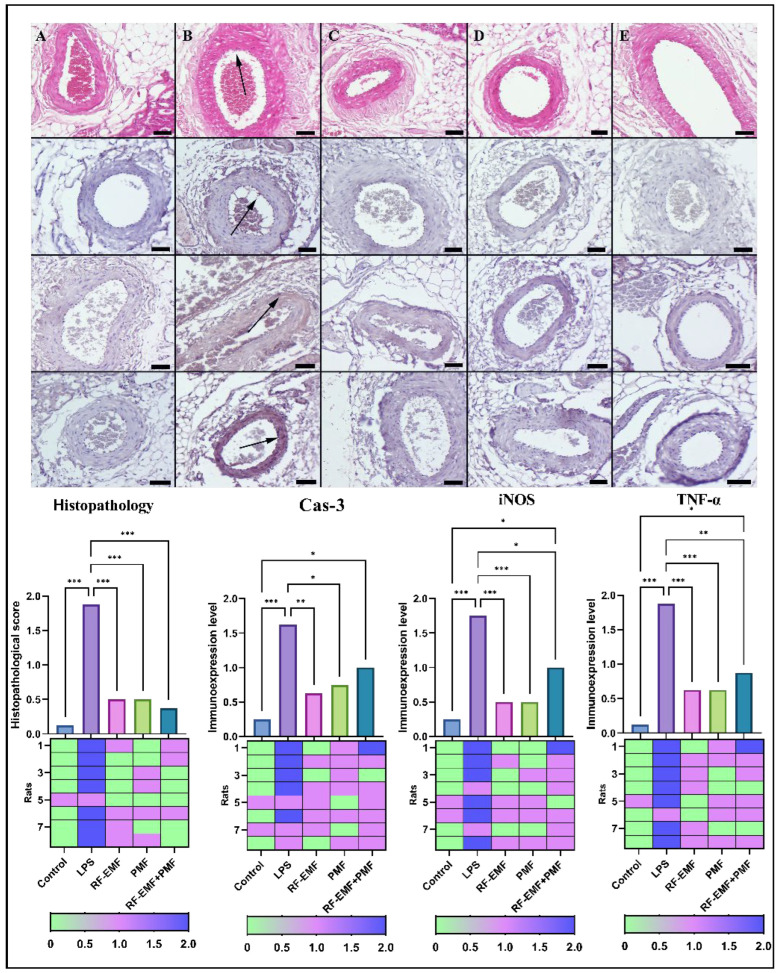
Histopathological (**top** row) and immunohistochemical analysis of caspase-3 (**second** row), iNOS (**third** row), and TNF-α (**fourth** row) expression in renal arteries across group. (**A**) Control group: Normal arterial histology with negligible caspase-3, iNOS, and TNF-αexpression. (**B**) LPS group: Evident endothelial shedding and significantly increased expression of caspase-3, iNOS, and TNF-α (arrows). (**C**) PMF group: Restored normal arterial histology and notable reduction in caspase-3, iNOS, and TNF-α (arrows). (**D**) RF-EMF group: Normal arterial appearance with decreased caspase-3, iNOS, and TNF-α expression. (**E**) Combined PMF + RF-EMF group: Preserved normal arterial histology and significantly reduced caspase-3, iNOS, and TNF-α expression, compared to control levels. The top row shows HE staining, and the remaining rows display results of the streptavidin-biotin peroxidase method; Scale bars = 50 μm. RF-EMF: radiofrequency electromagnetic field, PMF: pulsed magnetic field, Cas-3: caspase-3, iNOS: inducible nitric oxide synthase, TNF-α: tumor necrosis factor-alpha. Data are expressed as means ± standard deviations. Statistical significance was assessed using one-way ANOVA: * *p* < 0.05, ** *p* ≤ 0.01 *** *p* ≤ 0.001.

Normal vascular histology was observed in the control group. In rats treated with LPS, shedding of the endothelium in the renal arteries and hemorrhages around the vessels in some places was noted. Notably, PMF and RF-EMF applications preserved the structure of the vessels in both single and double applications. When the expression of Cas-3, iNOS, and TNF-α in the vessels was examined, it was determined that the expression that was negative or very light in the control group increased in the LPS group, and PMF and RF-EMF applications had a corrective effect on the expression in both single and combined applications (Figure 4).

At the immunohistochemical examination of the renal arteries revealed that TNF-α, Cas-3, and iNOS scores were significantly greater in the LPS group than in the control group (*p* < 0.001). When the scores of the control group and the treatment groups were compared, there was an increase in the scores of all three treatment groups, but only the increases in the TNF-α, Cas-3, and iNOS scores of the RF-EMF+PMF group were statistically significant (*p* < 0.05). When the scores of the LPS group and the treatment groups were compared, a decrease was observed in the scores of all three treatment groups [(Cas-3: from 1.62 ± 0.51 to 0.75 ± 0.46 (PMF), 0.62 ± 0.51 (RF-EMF), and 1.0 ± 0.53 (RF-EMF+PMF)], [(iNOS: from 1.75 ± 0.46 to 0.5 ± 0.53 (PMF), 0.5 ± 0.53 (RF-EMF), 1.0 ± 0.53 (RF-EMF+PMF)], [(TNF-α: from 1.87 ± 0.35 to 0.62 ± 0.51 (PMF), 0.62 ± 0.51 (RF-EMF), 0.87 ± 0.64 (RF-EMF+PMF)] (*p* < 0.05). No significant difference was found between the treatment groups (*p* > 0.05) (Figure 4).

### 3.2. Biochemical Findings

**Figure 5:**
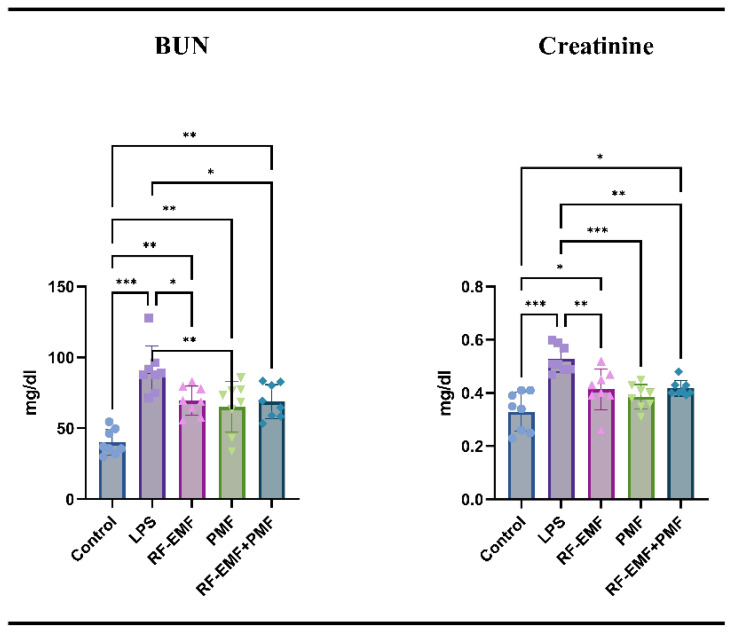
Serum levels of BUN and creatinine. LPS: Lipopolysaccharide, RF-EMF: Radiofrequency Electromagnetic Field, PMF: Pulsed Magnetic Field. The values are presented as the means ± SDs. The relationships between groups and the results of the biochemical marker analysis were assessed via one-way ANOVA. * *p* < 0.05, ** *p* < 0.01, *** *p* < 0.001.

When the serum BUN and creatinine values were compared between the groups, the values in the LPS group were significantly greater than those in the control group (*p* < 0.001). Compared to those in the control group, the biochemical values of all three treatment groups were greater (*p* < 0.05). In addition, except for the creatinine value in the PMF group, the increase in the values in the treatment groups compared to those in the control group was significant (*p* < 0.05). In all treatment groups, values were significantly lower than in the LPS group [(BUN: from 90.84 ± 17.08 mg/dl to 65.23 ± 18 (PMF), 69.54 ± 10.18 (RF-EMF), and 68.87 ± 11.89 (RF-EMF+PMF)] (*p* < 0.01; *p* < 0.05; *p* < 0.05, respectively), [(creatinine: from 0.52 ± 0.05 mg/dl to 0.38 ± 0.04 (PMF), 0.41 ± 0.07 (RF-EMF), and 0.41 ± 0.02 (RF-EMF+PMF)] (*p* < 0.001; *p* < 0.01; *p* < 0.01, respectively). No significant difference was observed between the treatment groups (*p* > 0.05) (Figure 5).

Although the TAS value was greater in the control group than in the LPS group, the difference was not statistically significant (*p* > 0.05). Compared to those in the LPS group, the TAS values were greater in the treatment groups, and the increases in the RF-EMF and RF-EMF + PMF groups were statistically significant (*p* < 0.05). When the treatment groups were compared, no significant difference was detected in terms of the TAS values (*p* > 0.05).

**Figure 6:**
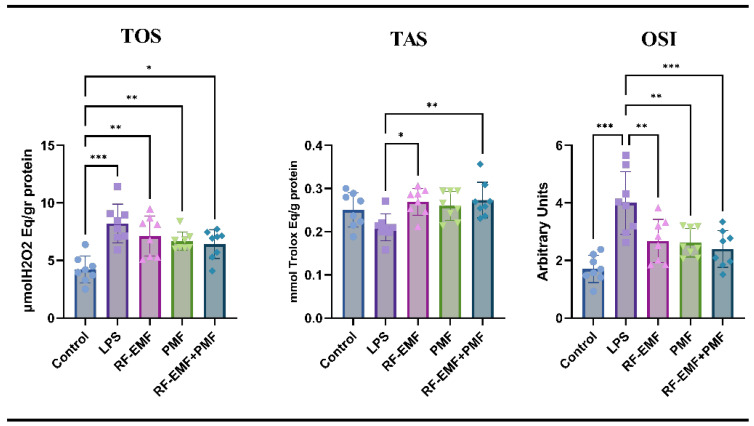
Oxidative stress parameters in this study. LPS: Lipopolysaccharide, RF-EMF: Radiofrequency electromagnetic field, PMF: Pulsed magnetic field, TOS: Total oxidant status, TAS: Total antioxidant status, OSI: Oxidative stress index. The values are presented as the means ± standard deviations, and Fisher’s test was used; * *p* < 0.05, ** *p* < 0.01, *** *p* < 0.001.

The TOS values were significantly greater in all the other groups than in the control group (*p* < 0.05). Although the TOS values of all three treatment groups were lower than those of the LPS group, the difference was not statistically significant (*p* > 0.05). When the treatment groups were compared, no difference was observed in terms of TOS values (*p* > 0.05). Although the OSI values were greater in all the groups than in the control group, the increase in the LPS group was significant (*p* < 0.001). The OSI values of all three treatment groups were significantly lower than those in the LPS group (from 4 ± 1.08 to 2.61 ± 0.49 (PMF), 2.67 ± 0.74 (RF-EMF), and 2.4 ± 0.63 (RF-EMF + PMF) (*p* < 0.01; *p* < 0.01; *p* < 0.001, respectively). When the treatment groups were compared, no statistically significant difference was observed in terms of OSI values (*p* > 0.05) (Figure 6).

### 3.3. Genetic Findings

Compared with those in the control group, increases in the IL-6, HIF-1α, Bax, and Cas-9 parameters and decreases in the eNOS and Bcl-2 parameters were observed in the LPS group (*p* < 0.001).

When IL-6 expression was compared between the groups, no significant difference was detected between the treatment groups and the control group (*p* > 0.05). The IL-6 values in the treatment groups were lower than those in the LPS group (from 3.4 ± 0.4 to 1.14 ± 0.05 (PMF), 1.3 ± 0.18 (RF-EMF), and 1.07 ± 0.08 (RF-EMF + PMF) (*p* < 0.001). When the treatment groups were compared, no significant difference was detected (*p* > 0.05).

**Figure 7:**
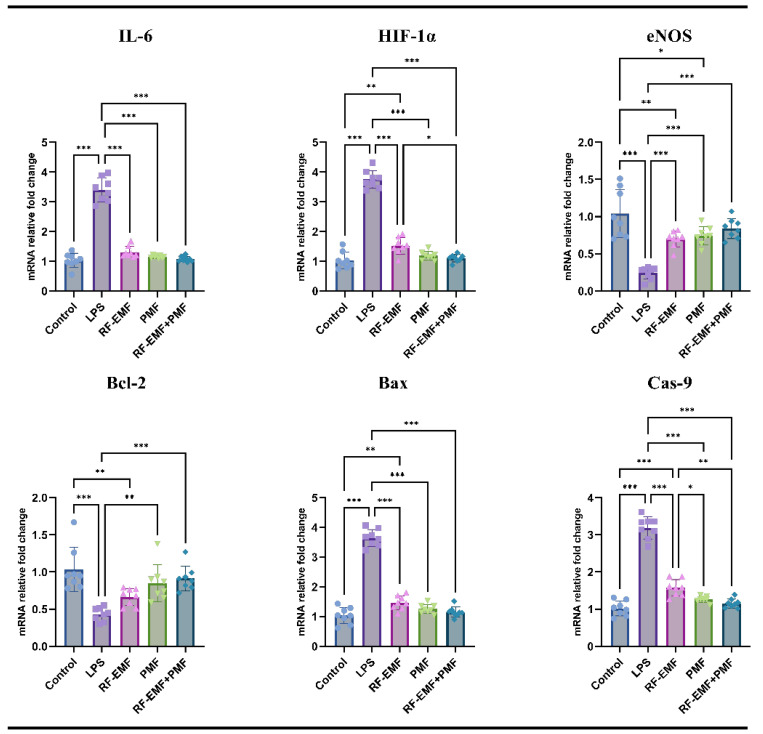
Relative mRNA expression results from genetic analysis, highlighting changes in the expression of key genes across groups. The graph depicts the expression levels of IL-6 (interleukin 6), HIF-1α (hypoxia-inducible factor 1α), eNOS (endothelial nitric oxide synthase), Bcl-2 (B-cell lymphoma 2), Bax (Bcl-2-associated X protein), and Cas-9 (caspase 9) under different experimental conditions. RF-EMF: radiofrequency electromagnetic field; PMF: pulsed magnetic field. Results are expressed as the means ± standard deviations. Statistical significance was evaluated using one-way ANOVA: * *p* < 0.05, ** *p* ≤ 0.01 *** *p* ≤ 0.001.

Although HIF-1α expression was greater in the RF-EMF group than in the control group (*p* < 0.01), the HIF-1α values in the PMF and RF-EMF + PMF groups were similar to those in the control group. The HIF-1α levels in the treatment groups were lower than those in the LPS group (from 3.75 ± 0.3 to 1.18 ± 0.14 (PMF), 1.52 ± 0.27 (RF-EMF), and 1.1 ± 0.12 (RF-EMF + PMF) (*p* < 0.001). The HIF-1α value in the RF-EMF group was greater than that in the other treatment groups, and the difference with the combined treatment group (RF-EMF + PMF), the HIF-1α value in the RF-EMF group was statistically significant greater than the combined treatment group (*p* < 0.05) (Figure 7).

When eNOS expression was compared with that in the control group, it was found to be low in all three treatment groups; the decrease in the RF-EMF and PMF groups was statistically significant (*p* < 0.05). The eNOS values in the treatment groups were greater than those in the LPS group (from 0.24 ± 0.08 to 0.74 ± 0.12 (PMF), 0.69 ± 0.1 (RF-EMF), and 0.84 ± 0.13 (RF-EMF + PMF) (*p* < 0.001). When the treatment groups were compared, no significant difference was detected (*p* > 0.05) (Figure 7).

Although Bcl-2 expression was lower in the RF-EMF group than in the control group (*p* < 0.01), there was no significant difference between the PMF and RF-EMF + PMF groups and the control group (*p* > 0.05). When the treatment groups other than the RF-EMF group were compared with the LPS group, the Bcl-2 values were significantly different (from 0.43 ± 0.09 to 0.84 ± 0.24 (PMF) and 0.91 ± 0.16 (RF-EMF + PMF) (*p* < 0.01).

Although Bax expression was greater in the RF-EMF group than in the control group (*p* < 0.01), no significant difference was found between the PMA and RF-EMF + PMF groups and the control group (*p* > 0.05). The Bax values in the treatment groups were lower than those in the LPS group (from 3.64 ± 0.28 to 1.25 ± 0.15 (PMF), 1.46 ± 0.23 (RF-EMF), and 1.15 ± 0.17 (RF-EMF + PMF) (*p* < 0.001). Although the Bax values in the RF-EMF group were greater than those in the other treatment groups, the differences were not significant (*p* > 0.05). Cas-9 expression was greater in the RF-EMF group than in the control group (*p* < 0.001); no significant difference was found between the PMF and RF-EMF + PMF groups and the control group (*p* > 0.05). Cas-9 values were lower in the treatment groups than in the LPS group (from 3.18 ± 0.29 to 1.26 ± 0.08 (PMF), 1.57 ± 0.22 (RF-EMF), and 1.15 ± 0.12 (RF-EMF + PMF) (*p* < 0.001). Although Cas-9 values were similar in the PMF and RF-EMF + PMF groups, lower Cas-9 values were observed in the RF-EMF group than in the other treatment groups (*p* < 0.05) (Figure 7).

## 4. Discussion

In this study, histopathological, immunohistochemical, biochemical, and genetic examinations revealed that both single and combined applications of 0.5 h RF-EMF and 3 h PMF reduced and even reversed SI-AKI in rats with sepsis induced by LPS.

Many studies in the literature have evaluated the therapeutic effects of magnetic fields on pathologies such as wound healing, pain in musculoskeletal disorders, osteoporosis, various types of cancer, and neurodegenerative diseases [12,13,14,17,26]. Magnetic fields were applied at different doses and durations in each of these studies, but there is no clear consensus on the appropriate dose and duration. In addition, unlike the pathologies mentioned above, sepsis is a disease that can develop within hours and can cause mortality within hours. For this reason, long-term magnetic field applications applied in other studies were not deemed appropriate for use in the sepsis model. There are few studies in the literature showing the effects of magnetic field application on sepsis [10,11]. In addition, our study is the first to evaluate the use of RF-EMF alone and in combination with PMF in the treatment of sepsis.

The biological effects of RF-EMF and PMF are highly sensitive to changes in exposure parameters. For instance, the radical pair mechanism explains how magnetic fields influence free radical reactivity, modulating oxidative stress [27,28]. Similarly, ion channel modulation by RF-EMF affects intracellular signaling, with small variations in intensity or frequency leading to drastically different outcomes [16]. Additionally, feedback mechanisms with time delays, such as the antioxidant response to ROS, highlight the importance of optimizing exposure durations to avoid overactivation or adaptation. These considerations underscore the need for precise parameter optimization, as performed in this study. Although the frequency value and intensity we used in this study are quite low compared to the literature [16], a preliminary study was conducted to determine the most effective periods in which we aimed to show the protective effects of two different magnetic fields on the acute effects of sepsis. Even if the frequency used is extremely low, we anticipated that harmful effects may occur if the duration of the application is extended. Prolonged exposure to RF-EMF or PMF can lead to the adaptation or overactivation of cellular pathways, reducing efficacy [16]. This is why exposure times are carefully optimized, as seen in this study (0.5 h for RF-EMF and 3 h for PMF). The periods used in the preliminary study were designed according to the 6 h required to create an acute sepsis model with LPS. The results of the preliminary study revealed that 3 h for PMF and 0.5 h for RF-EMF had the most ideal effect. The optimum periods determined in the preliminary study were used in the main study.

The pathophysiology of sepsis-associated AKI is complex and multifactorial. It includes hemodynamic changes in the kidney, endothelial dysfunction, inflammatory cell infiltration into the renal parenchyma, intraglomerular thrombosis, and obstruction of the tubules by necrotic cells and debris [29,30]. On the other hand, damage to the renal arteries and veins may restrict the blood flow of the tissue, leading to insufficient oxygen required by the cellular metabolism [31]. In light of this information, inflammation, oxidative stress, and apoptosis in the renal artery can also be evaluated as indicators of damage that will occur in the renal tissue [31,32]. With this foresight, the histopathological and immunohistochemical analyses we performed on the renal tissue in our study were also used in the renal artery. In the histopathological examination performed in our study, severe hyperemia and microhemorrhages were observed in the LPS group, whereas the kidneys and arteries in the treatment groups had a histological appearance similar to that of the control group. In a study aimed at evaluating the effects of PMF treatment on survival and organ damage in mice with LPS-induced septic shock, PMF treatment downregulated the proinflammatory cytokine expression induced by LPS in the liver, spleen, lung, and kidney tissues and contributed to survival by reducing histological organ damage [10].

Oxidative stress plays an important role in the development of sepsis-induced multiple-organ damage, which may develop directly or secondary to inflammation [33]. In pathogen-related inflammation, ROS, which are produced significantly during the phagocytic infiltration of neutrophils and macrophages, constitute a part of the innate immune response. It is balanced by antioxidant enzymes under physiological conditions. Excessive inflammatory responses caused by sepsis, endothelial damage, impaired vascular permeability and tissue perfusion, and excessive ROS production due to mitochondrial dysfunction disrupt the balance [34]. In our study, compared to those in the control group, the decreases in the TAS values in the LPS group were not significant, whereas the increases in the TOS and OSI values were significant. It is thought that the OSI, which is the ratio of TOS and TAS values to each other rather than evaluating them alone, provides more accurate results in showing the relationships between other variables and oxidative stress. The fact that the OSI values were significantly lower in all the treatment groups than in the control groups when the LPS and treatment groups were compared supports this finding [35].

Compared to other organs, the kidneys are highly susceptible to endothelial damage during sepsis [36]. Endotoxins from bacteria activate inflammatory cells and cause the release of proinflammatory cytokines (such as TNF-α and IL-6), which further exacerbate the inflammatory response. In LPS-mediated systemic inflammation, proinflammatory cytokines such as TNF-α, IL-1β, and IL-6 are known to play important roles in the pathogenesis of acute inflammation and renal vascular and parenchymal cell dysfunction [37,38]. In this study, increased IL-6, TNF-α, and iNOS levels in both the renal and arterial tissues of the injury group triggered inflammation in parallel with histopathological symptoms, and RF-EMF and PMF treatments reversed this phenomenon. The decreased cytokine levels in our findings show that the treatment groups were not superior to each other in terms of anti-inflammatory effects. Similar to this study, in two different studies conducted on BALB/c mice injected with LPS, 30 min of PMF application was shown to have a modulatory effect on hyperinflammatory response by reducing the levels of proinflammatory cytokines such as TNF-α and IL-6 [10,39].

The HIF-1α pathway is activated through oxidative stress caused by increased levels of IL-6 and mitochondrial ROS, which play critical roles in both changes in oxygen levels and the immune response and increase inflammation [37,40]. In this study, increased HIF-1α expression was observed in the injury group in parallel with increased proinflammatory cytokine levels compared to those in the control group, and decreased HIF-1α levels were observed in the treatment groups compared to those in the LPS group, indicating the positive effects of both separate and combined RF-EMF and PMF treatments on the resolution of inflammation.

Compared with most organs, the kidneys have a lower vascular tone, and NO plays a key role in maintaining normal vascular tone in the kidney [41]. Under basal conditions, NO released from the kidney contributes to the control of renal perfusion and participates in the regulation of renal hemodynamics, affecting renal vascular resistance [42]. NO is normally produced by eNOS, the structural isoform of NO synthase in the endothelium. This production may be inhibited as a result of vascular damage resulting from oxidative or inflammatory conditions; perfusion of the distal tissue may be impaired, and other damage mechanisms, including apoptosis, may be activated. In this study, the reduction in increased iNOS levels, i.e., inflammation, in the injury group induced by RF-EMF and PMF reversed the decrease in eNOS expression and maintained its levels. On the other hand, these applications can increase the blood flow of the tissue by increasing eNOS expression in an isolated manner and therefore reduce the oxidative stress and inflammation occurring in the tissue. In their study, Miura et al. reported that PMF application increased microperfusion and oxygenation in the brain tissue of rats by increasing NO synthesis [43].

Apoptosis occurs when a cell loses its capacity to control functional disorders and the spread of damage, and its control depends on the balance of antiapoptotic and proapoptotic genes [44]. In particular, in the event of damage to mitochondria, which are organelles responsible for oxygenation and energy metabolism, the gene expression of proapoptotic proteins such as Bax, Cas-9, and Cas-3 increases, whereas the expression of Bcl-2, an antiapoptotic protein, is suppressed [45]. In the study conducted by Jin et al., the effect of irisin on SI-AKI was investigated, and they reported that LPS increased Bax activity together with TNF-α and IL-1β, whereas Bcl-2 activity decreased [46]. Similarly, in a study investigating the effects of protopine on the LPS-induced SI-AKI model in BALB/c mice, decreased Bcl-2 levels were observed despite increased Bax, Cas-3, and Cas-9 levels in mice treated with LPS [47]. In this study, consistent with the literature, Bax and Cas-9 expression levels were found to be high, and Bcl-2 expression levels were found to be low in the genetic analyses of the injury group. In the treatment groups, Bax and Cas-9 expression was low, and Bcl-2 levels were high. The fact that the immunoexpression of Cas-3, which is the end product of this pathway, was found to be parallel to the genetic analyses proves that RF-EMF and PMF have antiapoptotic effects. The emergence of this effect may be caused by increased eNOS expression and decreased oxidative stress and inflammation.

BUN and creatinine are normal metabolic waste products excreted by the kidney, and their measurement is directly related to the excretory function of the kidney and is one of the most commonly used methods to evaluate kidney function today [48]. In this study, the increased BUN and creatinine values in the LPS group significantly decreased in all the treatment groups, indicating that the RF-EMF and PMF treatments may effectively improve kidney function. Despite this, the high values of the BUN and creatinine levels in the treatment groups compared to those in the control group may be because magnetic field treatments are more meaningful as additional treatment methods to other treatment options in the treatment of SI-AKI to fully restore renal function to normal levels or because the experimental period of 6 h, which is shorter than the half-life of creatinine and BUN, may not be sufficient time to achieve full recovery in biochemical analyses.

## 5. Conclusions

In conclusion, noninvasive, nontoxic, low-cost PMF and RF-EMF, both single and combined, have been demonstrated to have renoprotective anti-inflammatory, antioxidant, and antiapoptotic effects. We believe that this experimental study, which is promising for use as an alternative treatment method in the treatment of SI-AKI, may shed light on future advanced research.

## Figures and Tables

**Table 1:** Description of histopathological scores and the severity of the immunohistochemical re-action.

Score	Histopathological Grade	Immunohistochemical Grade
0	No damage	Negative
1	Mild hyperemia, slight hemorrhage, inflammation, and no degeneration	Focal weak staining
2	Severe hyperemia, slight hemorrhage, slight inflammation, and slight necrosis	Diffuse weak staining
3	Severe hyperemia, severe hemorrhage, marked inflammation, and marked necrosis	Diffuse strong staining

**Table 2:** Primary sequences, product sizes, and accession numbers of genes.

Genes	Primary Sequence	Product Size	Accession Number
*eNOS*	F: GGTTGACCAAGGCAAACCAC	247 bp	NM_021838.2
	R: CCTAATACCACAGCCGGAGG		
*Bcl-2*	F: CATCTCATGCCAAGGGGGAA	284 bp	NM_016993.2
	R: TATCCCACTCGTAGCCCCTC		
*Bax*	F. CACGTCTGCGGGGAGTCAC	419 bp	NM_017059.2
	R: TAGAAAAGGGCAACCACCCG		
*Cas-9*	F: AGCCAGATGCTGTCCCATAC	148 bp	XM_039110693.1
	R: CAGGAACCGCTCTTCTTGTC		
*IL-6*	F: CACAAGTCCGGAGAGGAGAC	168 bp	NM_012589.2
	R: ACAGTGCATCATCGCTGTTC		
*HIF-1α*	F: GCAACTAGGAACCCGAACCA	251 bp	NM_024359.2
	R: TCGACGTTCGGAACTCATCC		
*GAPDH (Housekeeping)*	F: AGGTTGTCTCCTGTGACTTC	130 bp	NM_017008.4
	R: CTGTTGCTGTAGCCATATTC		

*eNOS*: Endothelial nitric oxide synthase, *Bcl-2*: B-cell lymphoma 2, *Bax*: Bcl-2-associated X protein, *Cas-9*: Caspase 9, *IL-6*: Interleukin 6; *HIF-1α*: Hypoxia-inducible factor-1 alpha; *GAPDH*: Glyceraldehyde-3-phosphate dehydrogenase.

## Data Availability

The raw data supporting the conclusions of this article will be made available by the authors on request.

## Abbreviations

The following abbreviations are used in this manuscript:

LPS	Lipopolysaccharide
AKI	Acute Kidney Injury
SA-AKI	Sepsis-Associated Acute Kidney Injury
RF-EMF	Radiofrequency Electromagnetic Field
PCB	Printed circuit board
PMF	Pulsed Magnetic Field
TAS	Total Antioxidant Status
TOS	Total Oxidant Status
OSI	Oxidative Stress Index
IL-6	Interleukin-6
HIF-1α	Hypoxia-Inducible Factor-1 Alpha
NADPH	Nicotinamide adenine dinucleotide phosphate
NO	Nitric oxide
eNOS	Endothelial Nitric Oxide Synthase
Bcl-2	B-cell Lymphoma 2
Bax	Bcl-2-Associated X Protein
Cas-3	Caspase-3
Cas-9	Caspase-9
TNF-α	Tumor Necrosis Factor Alpha
iNOS	Inducible Nitric Oxide Synthase
GAPDH	Glyceraldehyde-3-Phosphate Dehydrogenase
ARRIVE	Animal Research: Reporting in Vivo Experiments
PCR	Polymerase Chain Reaction
RT-qPCR	Reverse Transcription Quantitative Polymerase Chain Reaction
DAB	Diaminobenzidine
